# Knowing Me Knowing You—Mentorship in Hospital Placement

**DOI:** 10.1111/tct.70230

**Published:** 2025-10-21

**Authors:** Monika Kvernenes, Hilde Grimstad, Edvin Schei, Cathinka Thyness, Tim Dornan, Terese Stenfors

**Affiliations:** ^1^ Centre for Medical Education & Department of Clinical Medicine, Faculty of Medicine University of Bergen Bergen Norway; ^2^ Department of Public Health and Nursing, Faculty of Medicine and Health Sciences Norwegian University of Science and Technology Torgarden Norway; ^3^ Department of Global Public Health and Primary Care University of Bergen Bergen Norway; ^4^ Øya Primary Health Care Center Trondheim Municipality Norway; ^5^ Centre for Medical Education Queens University Belfast UK; ^6^ Department of Learning, Informatics, Management and Ethics Karolinska Institutet Solna Sweden

**Keywords:** clinical learning environment, faculty development, mentors, near‐peer, workplace‐based learning

## Abstract

**Background:**

Despite junior doctors' pivotal role as primary contacts for students in clinical placements, they are typically offered minimal or no mentor training, and their knowledge about how to facilitate student learning remains experience‐based. We developed a co‐mentoring program where junior doctors were offered training focused on student learning and agency, near‐peer support and the promotion of psychological safety.

**Methods:**

To evaluate the intervention, we collected qualitative data using interviews, e‐mail prompts and a focus group among 13 junior doctors across four cohorts of mentor training. No baseline data were included. Data were analyzed using thematic analysis. The research aimed to explore the junior doctors' experiences of transitioning from an unclear supervisory role to a defined mentor role with a relational focus.

**Results:**

Mentors felt that they were making a significant difference to the students' learning and that focusing on relationship building and belonging enabled entrustment. Furthermore, mentoring was demystified after the training regime. Finally, the concept of collective responsibility throughout the medical department emerged as a crucial element in the mentoring dynamic.

**Discussion:**

Respondents suggested that the intervention had shifted their attitudes: Participants realized that establishing contact and actively collaborating with the students, also beyond the mentor's primary team, were beneficial for both students and mentors without demanding additional sacrifices of time or energy. However, the initiative did not manage to change organizational structures or create motivation in mentors to pursue further pedagogic development, underscoring the need to address structural barriers and promote a growth‐oriented mindset among mentors.

## 1 Introduction

Preparing clinicians to effectively mentor medical students during their clinical placements presents a persistent challenge. While there are frequent reports of medical students feeling marginalized or perceiving the hospital workplace as an unsupportive learning arena [1, 2, 3], less focus has been placed on the clinicians themselves and the support they need to facilitate students' learning [4]. With increased focus on the integration of basic science and clinical learning in medical curricula, there is a growing need to train diverse groups of clinician‐educators who need to thrive in their role and at the same time offer affective, pedagogic, and organizational support for students [5, 6]. Research demonstrates that clinicians often struggle to meet the expectations associated with the educator role [7, 8, 9, 10, 11]. Their efforts are challenged by the structures and affordances of the workplace, which often favors health service functions over education, causing clinicians to lack time, incentives and motivation to take on educator functions [12, 13, 14, 15].

Junior doctors, including interns and early residents in this hospital, play a pivotal role in facilitating favourable learning conditions for students. They often assume the role of near‐peer educators or may formally be appointed as supervisors, mentors, or preceptors. Despite often being the medical students' primary contact persons during hospital clerkship, they are typically offered minimal or no educator training [16]. Faculty development initiatives for mentors often target experienced clinicians, and clinician‐educator training has been found to focus predominantly on teaching, clinical leadership, and scholarship/research skills, and less on how to develop as a mentor or the importance of facilitating a trusting social and emotional learning environment [4, 16]. Furthermore, a recent review emphasizes that the existing literature gives little attention to the training of staff to help students integrate into clinical teams [17].

There is a gap in the literature exemplifying how junior medical doctors can be supported as they transition from an informal to a more formalized role as mentors for medical students. Hence, this article presents a qualitative evaluation study of a newly implemented mentoring scheme, exploring mentorship from the perspective of the mentors themselves. The study may enhance our understanding of how junior doctors adapt to the role as clinician‐educators and how this adaptation is shaped by workplace affordances—the opportunities and constraints for learning and participation embedded in the clinical environment [18, 19]. This paper addresses the research question: *What are junior doctors' experiences with taking on a team‐based near‐peer mentor role with relational focus?*

*There is a gap in the literature exemplifying how junior medical doctors can be supported as they transition from an informal to a more formalized role as mentors for medical students*.

## 2 Methodology

### 2.1 Research Context and Key Challenges

In Norway, undergraduate medical education programs last for 6 years, leading to the obtainment of a medical licence with limited job options. An 18‐month combined hospital and primary care internship is required before candidates can apply for specialized residency training.

This study was set in a medium‐sized local teaching hospital, which serves a population of approximately 180,000 inhabitants. The hospital welcomes 20 medical students each semester for an 8‐week clinical rotation (4 weeks at the surgical department and 4 weeks at the internal medicine department). It is located 130 km from the university campus, which means that students go to live there for the rotation period. For the students, this rotation is their first encounter with the somatic hospital workplace. The purpose of the rotation is for students to gain practical experience with patient and family communication, clinical examination, assessment of symptoms and findings, differential diagnostic evaluations, and proposing investigations and treatments in real clinical settings. Guidelines stress that as much learning as possible should take place through active participation in the hospital's daily routines. A service card listing all the procedures and tasks the students must do defines the formal requirement.

Previous evaluations of the hospital placement indicate that students' learning experiences are mixed. Although students are excited to engage in patient contact, several challenges were frequently reported, including frequent rotations between wards and a lack of a named supervisor.

Approximately 15 new junior doctors are hired each semester at the hospital. As most of them only stay for the duration of their one‐year internship, the turnover is high. Some do, however, remain for an additional 4–5 years to continue their residency training towards becoming specialists.

### 2.2 The Mentor‐Group Intervention

When the initiative began, the support junior doctors offered to medical students were informal, and they received no preparation for this role. Our iteratively developed intervention was based on what was feasible within the set curriculum and the junior doctors' limited available time and incentives to embark on an extensive training program. Hence, in collaboration with more experienced clinician‐educators and hospital leaders, we developed and implemented a team‐based near‐peer mentoring program. We organized junior doctors in mentor pairs and assigned each pair responsibility for two students during their 4‐week placement in internal medicine. This was done to offer students a psychologically safe relationship with at least two physicians and to provide mentors with peer–mentor support. The teams of mentors and assigned students (groups of four people) were advised to meet weekly to reflect on learning experiences and discuss potential challenges. Additionally, attempts were made to schedule rotations so that students could work together with one of their mentors in the clinic.

*In collaboration with more experienced clinician‐educators and hospital leaders*, *we developed and implemented a team‐based near‐peer mentoring program*.

Mentors were offered a one‐day faculty development seminar to prepare them for their mentoring role. The seminar, based on key principles from the experience based learning model (ExBL) [5] and transformative faculty development [20] pivoted well‐known challenges in students' hospital placements such as student learning, agency and participation, affective support, and the concept of psychological safety [21]. Furthermore, we prioritized the mentors' well‐being and intentionally modelled the supportive behaviours we expected them to demonstrate in their interactions with students. Mutual support among co‐mentors, setting boundaries, making students feel welcome, and practical strategies for managing student interactions under time constraints were also addressed. The seminar contained highly interactive sessions, and junior doctors' experiences as learners were articulated.

Students were on day one welcomed to the hospital and the physical learning environment, connected with their mentor team, introduced to other hospital staff, helped to identify salient learning goals and opportunities in the clinic, and encouraged to take on an agentic student role. All physicians in the department were informed about the project through e‐mails, staff meetings and informal conversations with key stakeholders.

The intervention was repeated over four consecutive semesters (Spring 2022 to Fall 2023). Each semester, two new cohorts of students spent 4 weeks at the department of medicine. The trained junior doctors continued in their mentor role, while new recruits were trained to replace those who switched to other jobs. In total, 15 junior doctors participated as mentors consecutively across the four semesters. None of the mentors withdrew from the program. Those who did not continue as mentors did so due to changes in job postings.

### 2.3 Research Design and Data Collection

This exploratory qualitative study explored the mentors' experiences with participating in this new approach to student mentoring. Thirteen mentors, nine female and four male, contributed data for the analyses as indicated in Table 1. Data was gathered between April 2022 and December 2023 and within 1 month after each student placement period. We used multiple qualitative methods to capture a comprehensive understanding of participant experiences. We conducted nine in‐depth interviews and facilitated one focus group discussion with mentors to gain detailed insights into their perceptions of the mentoring experience and its implementation. We particularly enquired about how they experienced the mentor role, what affordances and challenges they encountered, and the changes they observed resulting from the intervention. Interviews were conducted in Norwegian by MK, HG, ES and CT using the same interview guide (Appendix 1) and lasted 35–60 min. In addition, mentors provided written responses to open‐ended ‘e‐mail questions’ in multiple rounds, resulting in 11 responses. These questions were administered by the project leader ES via direct e‐mail to the individual mentors. This method enabled participants to reflect on their experiences over time and provide asynchronous feedback.

**TABLE 1:** Type of data gathered among mentors during the intervention period. Grey box indicates that the respondent was a mentor that semester.

Participant	Spring 22	Fall 22	Spring 23	Fall 23
Mentor 1	E‐mail questions Individual interview	E‐mail questions	Focus group	Individual interview
Mentor 2	E‐mail questions Individual interview	E‐mail questions		
Mentor 3	E‐mail questions Individual interview			
Mentor 4	E‐mail questions Individual interview			
Mentor 5	E‐mail questions Individual interview			
Mentor 6	E‐mail questions Individual interview			
Mentor 7			Focus group	
Mentor 8		E‐mail questions		
Mentor 9		E‐mail questions		
Mentor 10		E‐mail questions		
Mentor 11				Individual interview
Mentor 12			Focus group	
Mentor 13			Individual interview

*We used multiple qualitative methods to capture a comprehensive understanding of participant experiences*.

### 2.4 Data Analysis

Based on interview transcripts and written records of the e‐mail questions, data was analysed following the six‐phase approach of reflexive thematic analysis [22]. This involved familiarizing ourselves with the data, generating initial codes, constructing and reviewing potential themes, refining and defining themes and producing a final analytic narrative. This method allowed for the identification of patterns and themes within the data while acknowledging the researchers' reflexivity in interpreting findings. In the initial round, MK, HG and TS, who are all experienced qualitative researchers, read and re‐read the data to become deeply familiar with the content before engaging in an iterative process of inductive coding and theme development. Initial codes were generated inductively and independently by each researcher, followed by collaborative efforts to refine and merge codes as new insights emerged. Based on comparisons and reflexive discussions, codes were then grouped into potential themes according to conceptual similarity and relevance to the research question. In these discussions, team reflexivity and interpretative depth were prioritized over coder agreement. In the next round, we invited the broader research team to engage with the preliminary themes, thus broadening the analytical lens. Following discussions, themes were refined and described with illustrative quotes from the transcripts. The translations of quotes from Norwegian to English were reviewed by multiple team members to ensure consistency and fidelity to the original meaning.

### 2.5 Ethics and Reflexivity

The work reported in this paper stems from a research arm of a larger quality improvement project designed to enhance the quality of medical students' clinical rotation in hospitals. The dual purpose of both implementing change and doing research required balancing sometimes contradicting interests, such as between methodology rigor and objectivity on one side and stakeholder buy‐in and pragmatic decision‐making on the other. All authors were part of the project group, and HG, ES and CT also participated as facilitators in the mentor training. The project leader (ES) was frequently present and visible in the work environment throughout the project period, fulfilling multiple roles as a motivator, educator, change agent and researcher.

*The work reported in this paper stems from a research arm of a larger quality improvement project designed to enhance the quality of medical students' clinical rotation in hospital*.

The project group members held various backgrounds as medical doctors representing different career stages and educational researchers with longstanding experience in medical education research. None of the project members were employed at the hospital. All mentors who participated in the intervention did so voluntarily. They were informed and agreed to share their experiences with us and consented to the data being used for research purposes. The Norwegian Agency for Shared Services in Education and Research (SIKT) granted ethical approval for the project (ref. nr. 118210).

## 3 Results

The results should be read in the light of how the intervention played out in practice, which emerged throughout the data collection: Apart from the initial introduction, there was a large variation in terms of how often mentors had the opportunity to schedule meetings in their mentoring teams; some met regularly, while others had only ad hoc communication without formal meetings. Due to scheduling issues, some mentors worked side by side with 'their' students on a daily basis, whilst others had more interaction with students who belonged to other teams. Some mentors met their students as a team, whilst others met them individually. The following themes represent the junior doctors' mentoring experiences.

*The results should be read in the light of how the intervention played out in practice, which emerged throughout the data collection*.

### 3.1 Motivated by Making a Difference

The mentors reported enjoying being a mentor and felt motivated by the opportunity to contribute to students' learning. They alluded to their own first encounters with hospital placements and highlighted how they now, as physicians, felt they could make a difference by making the introduction to clinical work a positive experience for the students. One informant said, ‘As a student, I missed information about what life as a junior doctor was like, so I conveyed that [to my students]’ (Mentor 6, spring 22). The mentors saw themselves as role models. Observing students' excitement about seeing, hearing and learning new things was considered rewarding for the mentors. One mentor talked about feeling proud and happy on students' behalf:

*Observing students' excitement about seeing, hearing*, *and learning new things was considered rewarding for the mentors*.

*When they succeeded in a task that they were actually scared to do, that … that kind of proud walk down the hall when it was done, that they made it. That is so positive. It's really great! […] I felt proud of them*. (Mentor 3, spring 22)

### 3.2 Investing in Relationship Building to Enable Entrustment

Mentors highlighted that they were attentive to creating a friendly learning climate by welcoming the students to the workplace, memorizing their names, lowering the threshold for asking questions, and communicating clearly what was expected of the students. Mentors reported that staying in brief, yet repeated contact with students throughout the rotation, mostly through informal conversations, had a positive impact on their interactions. It allowed for better communication, students committing more to the chores they were given and mentors learned when they could entrust the students to do real patient work. One mentor said, ‘When you get to know them, you know that they can do certain tasks independently.’ (Mentor 7, spring 23). As a consequence, mentors felt students worked more independently and as assistant colleagues, thus helping out more than burdening their own work: ‘They are of great help to me, especially if they get to stay on for a few days you can see how they progress.’ (Mentor 11, fall 23).

Mentors underscored that having students in the department still required time and effort, but that the new approach involved investing that time in ways that were mutually beneficial for both the students and the mentors:

*I was surprised how little is needed for a student to learn more and be happier*. *Such as introducing them, just getting to know them as a person. And how much they can actually learn and then help us*. (Mentor 3, spring 22)

### 3.3 Demystifying Mentoring

Given that most mentors had limited prior experience with being mentored themselves, the roles and responsibilities associated with mentorship were initially unclear to many junior doctors. This uncertainty was compounded by a prevailing perception of students as an additional burden, as illustrated by one participant's comment: *‘*I think that, like everyone else, we are overworked. And when something comes up that initially feels like an additional task, it's easy to reject or ignore them.’ (Mentor 1, fall 23). However, after participating in mentor training and gaining hands‐on experience, many mentors reported finding the role unexpectedly gratifying. Most participants expressed feeling more confident in their mentoring abilities and having a clearer understanding of their responsibilities.

*The course day was good for describing what was expected from us and how we could actually do it. We became more confident that we are not just supposed to ‘serve’ the students but that we can challenge them, show our own uncertainties, that it's okay if we do not have time, and that we should not take on too much*. (Mentor 7, spring 23)

Mentors emphasized that establishing mentor‐mentee relationships, aligning mutual expectations, and fostering psychological safety were key factors in succeeding as mentors. Some mentors reported benefitting from having a co‐mentor to discuss and share the responsibility with.

### 3.4 Individual and Collective Responsibility

Mentors reported that although they had responsibility for two specific students in their team, they had as much contact with other students doing the rotation as they had with their assigned students. They explained that busy schedules and work rotations often prevented students and mentors from working side by side or together at all. Some reported that they only met with ‘ their’ students once or twice during the entire rotation. In spite of this, the mentors reported that there was a collective sense of responsibility among the mentors. Several informants reported that although only a few junior doctors were trained in the intervention, they had noticed a change both among students and staff towards being more ‘prepped’ than before.

*There was a collective sense of responsibility among the mentors*.

*I have been just as involved with students who are not “mine*.” *And, that has been a good thing, to be able to support each other's students across … or between mentor groups. The whole staff has become more aware*. (Mentor 1, spring 23)

## 4 Discussion

Based on an intervention focusing on forming mentoring groups, mentor training with inclusiveness, friendliness and feasibility and student agency as key ingredients, mentors reported positive intrinsic motivation and increased mastery in the mentoring role. They also experienced a change towards more collective responsibility for students. Finally, mentors identified the establishment of psychological safety as a useful approach for enabling students to engage in real‐patient learning [23].

*Mentors identified the establishment of psychological safety as a useful approach for enabling students to engage in real‐patient learning*.

Foundational to our intervention was a faculty development approach that focused on mentor‐mentee relationship building and mentors' learning and growth. The significance of affective support was role modelled through giving time and space in the seminar for the mentors to establish mutual trust, share learning experiences and by course facilitators caring for their well‐being. Our intention was that by experiencing a sense of belonging and of being entrusted with an important role, this message would transmit to the mentors' facilitation of students' learning. From our findings, we feel confident that this was successful. Furthermore, mentors reported a shift in attitude as they realized that establishing contact and actively collaborating with the students, also beyond the mentor's primary team, was beneficial for both students and mentors without demanding additional sacrifices of time or energy. However, our efforts to organize mentoring groups did not result in frequent meetings or close contact among group members throughout students' rotation.

*Our intention was that by experiencing a sense of belonging and of being entrusted with an important role, this message would transmit to the mentors' facilitation of students' learning*.

In our project, we experienced challenges in implementing major structural changes with the clinical workplace. Although rotations were prolonged, allowing students to familiarize themselves with personnel and routines on each ward, our efforts to align students' and mentors' work schedules, which would have allowed closer contact, were ultimately unsuccessful. Nevertheless, our findings suggest that mentors still experienced the group mentorship model as effective, and that it facilitated social connections between mentors and students, which in turn enabled students to be entrusted with clinical work. This indicates that the quality of the mentor–student relationship may have outweighed the quantity of time spent together. Furthermore, it suggests that the relational emphasis embedded in the mentor training may have had a ripple effect, positively influencing the learning environment beyond the individual relationships.

*The relational emphasis embedded in the mentor training may have had a ripple effect, positively influencing the learning environment beyond the individual relationships*.

Interestingly, the mentors participating in our study did not express the need nor motivation to further enhance their mentoring skills beyond what was offered. Reflecting on this, we believe this lack of growth mindset may result from our efforts to make mentoring manageable and, in the process, perhaps oversimplifying the complexity of the educational expertise embedded in the mentor role. By prioritizing feasibility over the ideal combination of high support and high challenge [24, 25], we may have risked modelling a behaviour we did not want the mentors to exhibit with the students. On the other hand, one might argue that as young doctors, a short introduction focusing on essential skills, learning environment and social inclusion may suffice to facilitate a positive sense of achievement. Moreover, the significance of offering junior doctors a mentoring experience that is both motivating and rewarding must be regarded as fundamental to their further development and the cultivation of their dawning identities as clinician educators [7, 11, 26].

Junior doctors often work on short‐term contracts before continuing their professional journeys towards residency in new locations, which challenges the sustainability of large‐scale mentor development. We therefore recommend mentor training to emphasize the mentors' motivation and confidence (to retain sustainability), clarifying mentor roles and expectations and the significance of social inclusion and belonging. Furthermore, we believe that it is important also to prime students, as highlighted in recent research [27]. The mentorship program has been continued in the teaching hospital in question. Future research exploring the long‐term effects from multiple stakeholders is welcomed, with particular attention to the medical students' perspective and the impact on quality improvement initiatives.

## 5 Methodological Reflections

Because of the nature of the project, being a quality development initiative with an added research layer, we recognize that our findings may have been influenced by the relationships that were established during the 3‐year long project period. To compensate, the analyses were mainly conducted by researchers who had little or no professional connection to the participants and primarily knew the project through the data itself. On the other hand, what was achieved in the project is unlikely to have transpired without the social and professional presence of change agents acting as motivators and supporters while diligently working to secure stakeholder buy‐in and collaboration. While data collection took place continuously throughout the two‐year intervention, no baseline data were included in the study. Furthermore, the findings presented are based on a single‐site study. To support assessment of transferability, we have provided contextual details that enable readers to consider the relevance of the described challenges, setting and outcomes to their own contexts.

## 6 Conclusions

Lack of time and resources in hospital workplaces calls for alternative bite‐sized faculty development initiatives that help clinician educators offer foundational support to students. By helping junior doctors invest in relationships with students and providing affective support through mentorship, the project laid the foundation for sustained motivation among junior doctors and hopefully for facilitating students' real‐patient learning. By offering a transparent description of both the important gains and the key challenges of our intervention, we aim to stimulate similar efforts to enhance clinical learning environments for both junior doctors and students.

*By helping junior doctors invest in relationships with students and providing effective support through mentorship, the project laid the foundation for sustained motivation among junior doctors and hopefully for facilitating students' real‐patient learning*.

## Author Contributions

**Monika Kvernenes:** conceptualization, data curation, formal analysis, funding acquisition, investigation, methodology, project administration, writing – original draft preparation, writing – review and editing. **Edvin Schei:** project administration, funding acquisition, investigation, project lead, writing – review and editing. **Hilde Grimstad:** conceptualization, formal analysis, investigation, methodology, writing – original draft preparation, writing – review and editing. **Cathinka Thyness:** investigation, writing – review and editing. **Tim Dornan:** conceptualization, writing – review and editing. **Terese Stenfors:** conceptualization, data curation, formal analysis, methodology, project administration, writing – original draft preparation, writing – review and editing.

## Ethics Statement

The Norwegian Agency for Shared Services in Education and Research (SIKT) granted ethical approval for the project (ref. nr. 118,210).

## Conflicts of Interest

The authors declare no conflicts of interest.

## Email and Interview questions

**Email questions (two rounds)**

1. What have you enjoyed the most in your role as a peer mentor?

2. What have you enjoyed the least?

3. Have the students been helpful to you in any way by performing clinical tasks? Can you provide examples? Do you think there is more to gain in the next round?

4. Have you learned anything new or gained new insights from being a mentor?

5. Do you regret taking on the role of a mentor? Feel free to write about this, whether the answer is mostly no or mostly yes.

6. Is there anything you would do differently in the second round of mentoring?

7. Have you enjoyed having a peer mentor partner? In what way?

8. Did you find the training day for all mentors useful or motivating? What was particularly good, and what can we do better next time?

9. Would you like to receive more pedagogical training?

10. If there is anything else you would like to share with us, you can write it here.

**Interview guide for individual interviews and focus group**

1. You have now mentored two students for a total of 8 weeks; how has it been?

2. How do you feel when you hear that there are students in the department? How does it affect you and your work?

3. Can you tell about a positive experience with mentoring students that you have had during these weeks?

4. Have you had any negative experiences with mentoring students during these weeks?

5. How is it for you to have students by your side in your clinical work? Does it evoke any feelings? Have any dilemmas arisen?

6. The way forward: what do you need now? What would you like to learn more about?
